# Analysis of *VEGFA* Variants and Changes in VEGF Levels Underscores the Contribution of VEGF to Polycystic Ovary Syndrome

**DOI:** 10.1371/journal.pone.0165636

**Published:** 2016-11-15

**Authors:** Wassim Y Almawi, Emily Gammoh, Zainab H. Malalla, Safa A. Al-Madhi

**Affiliations:** Department of Medical Biochemistry, Arabian Gulf University, Manama, Bahrain; Peking University Third Hospital, CHINA

## Abstract

**Background:**

Vascular endothelial growth factor (VEGF) contributes to the pathogenesis of polycystic ovary syndrome (PCOS), and genetic variations in *VEGFA* gene were suggested to contribute to VEGF secretion and PCOS.

**Aim:**

To evaluate the association of altered VEGF levels, stemming from the presence of specific *VEGFA* variants, with altered risk of PCOS.

**Subjects and Methods:**

Retrospective case-control study, performed between 2012–2015. Study subjects comprised 382 women with PCOS, and 393 control subjects. ELISA measured VEGF levels; genotyping of *VEGFA* variants was done by allelic exclusion.

**Results:**

Among the 12 tested *VEGFA* SNPs, minor allele frequency of only rs3025020 was significantly higher in PCOS cases than control women. Increased and reduced PCOS risk was seen with rs3025020 and rs2010963 genotypes, respectively. Increases and reduction in VEGF levels were associated with rs3025020 and rs2010963, respectively. Increased fasting insulin and HOMA-IR, and bioactive testosterone were linked with rs3025020, while carriage of rs2010963 was linked with reduction in fasting insulin, and free and bioactive testosterone. Of the 12 *VEGFA* variants, 9 were in LD, thus allowing construction of 9-locus haplotypes. Increased frequency of CAACAGCGA haplotype was seen in PCOS cases, after controlling for BMI, free and bioactive testosterone, SHBG, free insulin and HOMA-IR.

**Conclusion:**

This study confirms the contribution of altered VEGF secretion, resulting from genetic variation in VEGFA gene into the pathogenesis of PCOS. This supports a role for VEGF as PCOS candidate locus.

## Introduction

Polycystic ovary syndrome (PCOS) is a common endocrine disorder, which is characterized by clinical and/or biochemical hyperandrogenism, anovulation, and polycystic ovarian morphology ([1, 2]. It affects around 5–18% of reproductive age women, making it the leading cause of infertility in women [1, 3]. Alongside the reproductive abnormalities, PCOS is linked with metabolic disturbances, which include obesity, insulin resistance, altered glucose tolerance, and dyslipidemia [2, 4, 5]. Although the exact pathogenesis of PCOS has yet to be established, there is growing evidence of genetic influences, as seen through familial clustering and twin studies, which showed a 71% concordance in monozygotic twins in one study [6, 7].

Vascular endothelial growth factor (VEGF) is heparin binding, homo-dimeric glycoprotein, which binds to its high affinity receptors on endothelial cells, mediating angiogenesis and vascular permeability [8], and is secreted by ovarian granulosa lutein cells and theca cells, and uterine endometrium [9]. VEGF plays a critical role in ovarian folliculogenesis and normal reproductive function [8], highlighted by the findings that women with PCOS had increased serum levels of VEGF, which paralleled increases in Doppler flow velocities within ovarian vessels [10, 11]. High vascularization may result in abnormal growth of the theca interna, the site of androgen steroidogenesis, leading to hyperandrogenism, a hallmark of PCOS [12, 13]. VEGF is encoded by *VEGFA* gene, which is located on chromosome 6 (6p21.3), and comprises eight exons separated by seven introns [14]. Several single nucleotide polymorphisms (SNPs) were identified within the *VEGFA* gene, of which some were functional, directly affecting VEGF secretion [15–17]. These include -2578C/A and -1154G/A (promoter), -583T/C (intron 6), -634G/C and +963C/T (5’-untranslated region), which were linked with decreased VEGF production [15, 17, 18], whereas -460T/C and +534C/T were associated with higher VEGF secretion [16, 18].

Few studies investigated the possible association between *VEGFA* variants and PCOS and associated features. Two studies conducted on Turkish [19] and South Indian [20] cohort revealed no association between -2578C/A and -460T/C *VEGFA* SNPs and PCOS. Other studies that investigated the association between *VEGFA* SNPs and adverse pregnancy outcomes, such as ovarian hyperstimulation syndrome, ovarian cancer [21], pre-eclampsia [22, 23], spontaneous miscarriage [24, 25], and cervical cancer [26] yielded mixed results. This study evaluated the association of *VEGFA* variants with altered VEGF level and PCOS in 382 Bahraini Arab women with PCOS, and 393 age- and ethnically-matched control women.

## Subjects and Methods

### Subjects

This study was done as per the Helsinki Declaration guidelines. Women with PCOS (n = 382) were recruited from adult endocrinology and obstetrics/gynecology outpatient clinics in Manama, Bahrain. In addition, 393 age- and ethnically-matched eumenorrheic university employees and students, as well as healthy volunteers, were included as controls, and were recruited from the five governorates of Bahrain, thus were representative of present-day Bahraini population. Control subjects were examined in the menstrual cycle follicular phase, and their testosterone levels were within range (0.4–3.5 nmol/L). PCOS diagnosis was based on the 2003 Rotterdam Criteria [1, 12], according to two of three conditions were to be met: ultrasound evidence of polycystic ovary, anovulation, and hyperandrogenism. Subjects were excluded if they had hyperprolactinemia, non-classical adrenal hyperplasia, androgen-producing tumors, 21-hydroxylase deficiency, Cushing's syndrome, and active thyroid disease.

Additional exclusion criteria body mass index (BMI) extremes (<18 kg/m^2^ or >45 kg/m^2^), recent/present illness, and treatment, which affect carbohydrate metabolism or hormonal levels, for three months or longer before inclusion the study. Women using anti-hypertensive, oral contraceptive, anti-inflammatory, and lipid-lowering drugs were excluded. Demographic information, along with detailed personal and family history of diabetes, hypertension, infertility, hypercholesterolemia, and ischemic heart disease were obtained from all participants. The Bahraini Ministry of Health and Arabian Gulf University Research and Ethics Committees approved the study protocol, and all participants gave written informed consent to participate.

### Biochemical analysis

Peripheral venous fasting blood samples were obtained at 7:00–9:00 am during the early follicular phase of the menstrual cycle (days 2–5) for control subjects or women with PCOS who did not present with menstrual irregularities (i.e. those diagnosed with PCOS based on hyperandrogenism and polycystic ovaries), or any day for women with PCOS with menstrual irregularities, after an overnight (> 12 h) fast. Serum samples were analyzed for SHBG by sandwich ELISA (R&D Systems, Minneapolis, MN); assay sensitivity was 0.01 nmol/ml, and inter-assay and intra-assay precision (CV%) were 5.3% and 4.3%, respectively. Serum LH, FSH, TSH, testosterone, and glucose (ADVIA Centaur, Bayer Vital, Fernwald, Germany), and insulin (IMMULITE 2000, DPC Biermann, Bad Nauheim, Germany), were measured by automated chemiluminescence immunoassays. Free (FT) and bioactive (BT) testosterone and free androgen index (FAI) were determined using Free & Bioavailable Testosterone Calculator (www.issam.ch/freetesto.htm). ‬ Insulin resistance (IR) was estimated by the homeostasis model assessment (HOMA-IR), defined as fasting serum insulin (μIU/ml) × fasting plasma glucose (mmol/L)/22.5.‬‬‬‬‬‬‬‬‬‬‬‬‬‬‬‬‬‬‬‬‬‬‬‬‬‬‬‬‬‬‬‬‬‬‬‬‬‬‬‬‬‬

Measurement of hs-CRP in plasma samples was done by latex-enhanced nephelometry on a BN II Nephelometer (Dade Behring, Milan, Italy). Samples were assayed in duplicate in each analytical run; the lower limit of detection was 0.15 mg/L, and the assay range was 0.175–11.0 mg/L (initial dilution). Samples were tested in duplicates for IL-6 (Cat. No. D6050), IL-10 (D1000B), and TNFα (Cat. No. DTA00C) levels by sandwich ELISA (R&D Systems, Minneapolis, MN). Assay sensitivity, inter-assay and intra-assay precision (CV%) were 0.7 pg/ml, 4.5% and 2.6% for IL-6, 3.9 pg/ml, 6.7% and 3.4% for IL-10, and 5.5 pg/ml, 7.6%, and 4.9% for TNFα, respectively.

### Serum VEGF Measurement

Serum was prepared by centrifugation of peripheral venous blood (2000 *g*, 10 min), and was stored in 200 μl aliquots at or below -40°C. Human VEGF ELISA (R&D Systems, Minneapolis, MN) was used to test for serum VEGF concentration. Assay sensitivity was 9 pg/ml, and intra-assay (4.5–6.7%) and inter-assay (6.2–8.8%) precision (CV %) were within acceptable range.

### *VEGFA* Genotyping

*VEGFA* polymorphisms with minor allelic frequency (MAF) of >5% in Caucasians were selected. Allelic discrimination method (VIC- and FAM-labeled) was used for *VEGFA* genotyping. Assay-on-demand TaqMan assays were ordered from Applied Biosystems (Foster City, CA): rs833052 (C_8311577_10), rs1547651 (C__8311590_10), rs699947 (C_8311602_10), rs833061 (C_1647381_10), rs1570360 (C_1647379_10), rs2010963 (C_8311614_10), rs25648 (C_791476_10), rs833068 (C_11400864_10), rs833070 (C_1647373_10), rs3025020 (C_1647366_10), rs3025026 (C_1647362_10), and rs3025039 (C_16198794_10). The reaction was carried out in a 6.3 μl volume according to the instructions of the manufacturer (Applied Biosystems), using both StepOne and StepOne Plus real-time PCR system. For quality control, replicate blinded specimens were tested to evaluate genotyping reproducibility; concordance exceeded 99%.

### Statistical Analysis

Statistical analysis was done using SPSS (version. 22.0, IBM). Continuous variables were presented as means (±SD) for, while categorical variables were expressed as percentages of total. Differences in means were assessed by Student’s *t*-test, while inter–group significance was evaluated by Pearson χ^2^ test (or Fisher’s exact test for small samples). Hardy–Weinberg equilibrium (HWE) was tested by Haploview (www.broad.mit.edu/mpg/haploview). Calculation of study power was done by Genetic Power Calculator (http://pngu.mgh.harvard.edu/~purcell/cgi-bin/cc2k.cgi); the parameters used were 383 women with PCOS and 393 control women, genotypic relative risk for heterozygote (1/2) and minor allele homozygous (2/2), and the MAF for PCOS cases and controls for the seven tested SNPs, and assuming a 14.0% population prevalence of PCOS (unpublished Bahrain Ministry of Health statistics). Assuming these parameters, we calculated the overall power (66.0%) as the average power of the 12 tested SNPs. All analyses were performed under the assumption of additive genetic effect. Haploview was used to check linkage disequilibrium (LD) between SNPs beside their haplotype patterns. *VEGFA* haplotypes was reconstructed by the expectation maximization method. Taking the control group as reference, regression analysis was used for determination of the odds ratios (OR) and 95% confidence intervals (95%CI) associated with PCOS risk.

## Results

### Study Subjects

The biochemical and clinical characteristics of women with PCOS and control subjects are presented in Table 1. Comparable age, menarche, waist-hip ratio, along with lipid profile, total testosterone, LH and FSH levels at examination, were seen between women with PCOS and control women. Significant differences between cases and controls were noted for mean BMI (*P* <0.001), free testosterone (*P* = 0.009), bioactive testosterone (*P* = 0.004), free androgen index (FAI; *P* = 0.015), fasting insulin (*P* <0.001), and the insulin resistance index HOMA-IR (*P* <0.001). Significantly lower levels of SHBG (*P* <0.001), and higher levels of TSH (*P =* 0.006) (though within reference range) were noted between women with PCOS and controls. Accordingly, we controlled for these covariates in later analysis.

**Table 1:** Baseline and endocrine parameters of women with PCOS and control women.

	Cases ^*1*^	Controls ^*1*^	*P* ^*2*^
Age (years) ^*3*^	29.8 ± 5.4	28.1 ± 6.5	0.338
BMI (kg/m^2^) ^*3*^	29.9 ± 6.3	25.7 ± 5.3	2.3×10^−6^
Waist/hip ratio (WHR) ^*3*^	0.85 ± 0.12	0.83 ± 0.12	0.286
LH (IU/L) ^*4*^	6.5 (0.8–66.6)	5.4 (0.4–56.3)	0.063
FSH (IU/L) ^*4*^	5.3 (0.5–16.8)	5.3 (0.4–15.4)	0.568
LH/FSH ^*4*^	1.3 (0.05–6.12)	1.0 (0.2–10.9)	0.060
TSH (μIU/ml) ^*3*^	2.7 ± 2.1	1.7 ± 0.9	0.006
Menarche (years) ^*3*^	12.4 ± 1.4	12.6 ± 1.4	0.247
Total testosterone (nmol/L) ^*3*^	1.8 ± 1.0	1.7 ± 1.1	0.583
Free testosterone (pmol/L)	25.2 (3.3–92.4)	18.4 (0.7–49.2)	0.009
Bioavailable testosterone (pmol/L) ^*4*^	684.0 (149.0–2020.0)	469 (95.9–1480.0)	0.004
Free androgen index (FAI) ^*4*^	5.1 (0.7–320.8)	3.1 (0.3–151.7)	0.015
SHBG (nmol/L) ^*4*^	20.1 (12.9–185.3)	57.7 (12.6–189.8)	1.4×10^−6^
Fasting insulin ^*4*^	10.2 (1.6–99.4)	7.2 (1.7–22.1)	2.6×10^−4^
HOMA-IR ^*3*^	3.8 ± 2.7	1.9 ± 0.9	1.8×10^−4^
Total cholesterol (mmol/L) ^*3*^	4.8 ± 1.3	4.7 ± 1.0	0.804
HDL-cholesterol (mmol/L) ^*3*^	1.3 ± 0.6	1.4 ± 0.5	0.498
LDL-cholesterol (mmol/L) ^*3*^	2.7 ± 0.9	2.6 ± 0.6	0.591
Triglycerides (mmol/L) ^*3*^	1.5 ± 1.0	1.1 ± 0.8	0.135
sVEGF ^*4*^	228.9 (2.5–2,523.5)	140.2 (2.5–3,956.0)	0.007

1. A total of 382 PCOS cases and 393 control women were included.

2. Student’s *t*-test (variables with normal distribution), Mann-Whitney U-test (variables that were not normally distributed).

3. Mean ± SD.

4. Median (range).

### *VEGFA* SNPs analysis

A total of 12 *VEGFA* SNPs were selected for this analysis based on their established MAFs of >5% in Caucasians. MAFs of the 12 tested *VEGFA* SNPs between women with PCOS and control women are presented in Table 2. All the SNPs conformed to HWE (*P* <0.005). MAF of rs3025020 (-583T>C) was higher in women with PCOS than control women (0.34 *vs*. 0.29; *P* = 9.0 × 10^−4^). The MAF of other 11 SNPs were not significantly different between women with PCOS and control subjects.

**Table 2:** *VEGFA* SNPs Analyzed.

SNP	Location ^*1*^	HWE *P*	Cases^*2*^	Controls^*2*^	χ^2^	*P* ^*3*^	aOR ^*4*^ (95% CI)	Power
rs833052	43723335	0.78	0.16	0.19	2.19	0.14		50.0
rs1547651	43730644	0.62	0.21	0.18	0.66	0.42		64.3
rs699947	43736389	0.48	0.41	0.42	0.17	0.68		57.7
rs833061	43737486	0.91	0.43	0.42	0.17	0.68		58.4
rs1570360	43737830	0.09	0.30	0.28	1.33	0.25		71.1
rs2010963	43738350	0.45	0.34	0.35	0.35	0.56		57.0
rs25648	43738977	0.46	0.16	0.18	0.42	0.52		72.9
rs833068	43742527	0.91	0.36	0.34	0.79	0.37		67.8
rs833070	43742626	0.41	0.41	0.45	1.43	0.23		78.4
rs3025020	43749110	0.56	0.34	0.29	11.07	9.0 × 10^−4^	2.33 (1.33–3.76)	100.0
rs3025036	43751669	0.16	0.33	0.33	0.01	0.97		53.3
rs3025039	43752536	0.78	0.12	0.10	0.85	0.36		61.1

HWE, Hardy-Weinberg Equilibrium.

1. Location on chromosome based on dbSNP build 125.

2. Minor allele defined based on frequency in controls.

3. Adjusted *P* value, adjusted for age and BMI.

### *VEGFA* Genotype distribution

Significant difference in rs3025020 (-583T>C), and to a lesser extent rs2010963 (-634C>G) genotype distribution was seen between PCOS cases and control women (Table 3). There was enrichment of homozygous minor allele carrying genotypes -583C/C in PCOS cases (0.14 *vs*. 0.09), and heterozygous -634C/G genotypes in control women (0.48 *vs*. 0.37) (Table 3). For the remaining variants, comparable distribution of genotypes was seen among women with PCOS and controls. Taking homozygous major allele genotype as reference, increased risk of PCOS was found to be associated with -583T/C [OR (95% CI) = 1.89 (1.09–3.26)], while reduced PCOS risk was seen with only -634C/G [OR (95% CI) = 0.65 (0.44–0.97)] genotypes, thus assigning PCOS susceptibility and protective nature to these genotypes, respectively.

**Table 3:** VEGFA Genotype Frequencies.

	1 / 1 ^*1*^				1 / 2 ^*1*^			2 / 2 ^*1*^		
SNP	Cases	Controls	*P*	OR (95%CI)	Cases	Controls	OR (95% CI)	Cases	Controls	OR (95% CI)
rs833052	280 (0.73) ^*2*^	264 (0.67)	0.47	1.00 (Ref.)	81 (0.21)	109 (0.28)	0.72 (0.37–1.40)	21 (0.05)	20 (0.05)	1.56 (0.40–6.03)
rs1547651	247 (0.65)	266 (0.68)	0.56	1.00 (Ref.)	109 (0.29)	113 (0.29)	1.03 (0.59–1.83)	26 (0.07)	14 (0.04)	1.88 (0.60–5.85)
rs699947	135 (0.35)	165 (0.42)	0.10	1.00 (Ref.)	183 (0.48)	178 (0.45)	1.25 (0.82–1.90)	64 (0.17)	50 (0.13)	1.56 (1.01–2.42)
rs833061	130 (0.34)	132 (0.34)	0.69	1.00 (Ref.)	174 (0.46)	190 (0.48)	0.93 (0.65–1.33)	78 (0.20)	71 (0.18)	1.12 (0.72–1.76)
rs1570360	197 (0.52)	218 (0.55)	0.54	1.00 (Ref.)	140 (0.37)	131 (0.33)	1.13 (0.72–1.79)	45 (0.12)	44 (0.11)	1.12 (0.64–1.96)
rs2010963	183 (0.48)	161 (0.41)	0.04	1.00 (Ref.)	142 (0.37)	190 (0.48)	0.65 (0.44–0.97)	57 (0.11)	42 (0.11)	1.18 (0.65–2.14)
rs25648	271 (0.71)	261 (0.66)	0.31	1.00 (Ref.)	97 (0.25)	123 (0.31)	0.75 (0.48–1.19)	14 (0.04)	9 (0.02)	1.66 (0.47–5.80)
rs833068	156 (0.41)	156 (0.40)	0.21	1.00 (Ref.)	175 (0.46)	200 (0.52)	0.88 (0.63–1.22)	51 (0.13)	34 (0.09)	1.40 (0.82–2.37)
rs833070	132 (0.35)	126 (0.32)	0.59	1.00 (Ref.)	183 (0.48)	184 (0.47)	0.95 (0.63–1.45)	67 (0.18)	83 (0.21)	0.76 (0.45–1.30)
rs3025020	179 (0.47)	203 (0.52)	0.06	1.00 (Ref.)	149 (0.39)	155 (0.39)	1.06 (0.75–1.51)	54 (0.14)	35 (0.09)	1.89 (1.09–3.26)
rs3025036	180 (0.47)	186 (0.47)	0.52	1.00 (Ref.)	154 (0.40)	158 (0.40)	1.04 (0.83–1.29)	48 (0.13)	49 (0.12)	1.08 (0.65–1.54)
rs3025039	296 (0.77)	318 (0.81)	0.39	1.00 (Ref.)	81 (0.21)	68 (0.17)	1.29 (0.87–1.90)	5 (0.01)	7 (0.02)	0.76 (0.23–2.52)

1. Genotypes were coded as per “1” = major allele, “2” = minor allele.

2. Number of subjects (frequency).

### VEGF serum levels and PCOS features

Mean serum VEGF levels were significantly increased (*P* = 0.007), and reduced (*P* = 0.048) among rs3025020 and rs2010963 genotype carriers, respectively (Table 4). There was progressive increase in fasting insulin (*P* = 0.033) and HOMA-IR (*P* = 0.050), and bioactive testosterone (*P* = 0.042) in rs3025020 genotype carriers. On the other hand, significant reduction in fasting insulin (*P* = 0.012), free testosterone (*P* = 0.007) and bioactive testosterone (*P* = 0.001) in rs2010963 genotype carriers (Table 4).

**Table 4:** VEGF serum levels and PCOS features according to rs3025020 and rs2010963 genotypes ^1^.

	rs3025020				rs2010963			
Parameter	C/C	C/T	T/T	*P* ^*2*^	C/C	C/G		G/G	*P* ^*2*^
sVEGF	277.2±163.1	325.1±215.1	538.4±260.6	0.007	471.8±233.4	349.1±228.0	319.7±201.4	0.048
BMI	28.1 ± 7.1	28.6 ± 6.0	29.5 ± 6.9	0.396	29.1 ± 7.1	27.7 ± 5.9	27.3 ± 6.1	0.214
WHR	0.96 ± 0.10	0.94 ± 0.09	0.95 ± 0.11	0.197	0.95 ± 0.09	0.94 ± 0.09	0.95 ± 0.12	0.467
Menarche	12.5 ± 1.3	12.4 ± 1.4	12.4 ± 1.8	0.794	12.4 ± 1.5	12.3 ± 1.2	12.9 ± 1.7	0.476
Fasting insulin	13.7 ± 9.3	16.1 ± 11.4	17.1 ± 12.4	0.033	17.6 ± 11.5	16.4 ± 12.9	14.1 ± 11.5	0.012
SHBG	34.0 ± 24.0	32.7 ± 15.3	29.4 ± 15.2	0.385	34.2 ± 24.2	34.6 ± 20.3	35.4 ± 19.9	0.123
HOMA-IR	3.35 ± 2.34	3.96 ± 2.81	4.48 ± 3.26	0.050	4.65 ± 2.39	4.47 ± 3.52	3.28 ± 2.54	0.097
Total testosterone	1.28 ± 0.82	1.40 ± 1.01	1.58 ± 0.83	0.311	1.54 ± 0.81	1.38 ± 0.86	1.37 ± 0.84	0.428
Free testosterone	37.2 ± 22.3	28.0 ± 19.5	29.7 ± 19.6	0.169	31.3 ± 22.6	27.6 ± 17.9	25.9 ± 15.1	0.007
Bioactive testosterone	0.60 ± 0.42	0.72 ± 0.49	0.79 ± 0.42	0.042	0.68 ± 0.49	0.64 ± 0.44	0.60 ± 0.38	0.001
FSH	5.3 ± 1.4	5.7 ± 2.5	5.5 ± 2.2	0.647	5.9 ± 2.2	5.8 ± 1.9	5.3 ± 2.0	0.861
LH	7.8 ± 2.7	7.7 ± 1.8	7.0 ± 1.9	0.465	8.0 ± 1.9	7.6 ± 1.8	7.5 ± 1.6	0.805

1. Determined by ELISA; results expressed as pg/ml.

2. One-way ANOVA.

### Correlation studies

Correlation analysis were used to investigate the relationship between *VEGFA* rs30250202 and rs2010963 genotypes and PCOS-associated features; separate analyses were performed for each of the two *VEGFA* SNPs (Table 5). In the model including rs3025020, carriage of the minor (C) allele was positively associated with hs-CRP. On the other hand, in the model including rs2010963, there was a negative association between the presence of the minor allele (C) with erythrocyte sedimentation rate (ESR), but not with hs-CRP. There was no correlation between either rs3025020 or rs2010963 with the extent of obesity or hypertension, with oligomenorrhea, or with altered levels of (anti-inflammatory) IL-10 or (pro-inflammatory) IL-6 and TNFα.

**Table 5:** Correlation between rs3025020 and rs2010963 and obesity, hypertension, oligomenorrhoea, and inflammatory markers.

	rs3025020		rs2010963	
Parameter	Kendall's tau-b	Spearman’s rho	Kendall's tau-b	Spearman’s rho
Obesity (≥ 30 kg/m^2^)	0.041 (0.455) ^*1*^	0.046 (0.462) ^*2*^	0.021 (0.672)	0.025 (0.670)
Hypertension	0.113 (0.070)	0.119 (0.070)	-0.043 (0.403)	-0.050 (0.404)
Oligomenorrhoea	0.044 (0.345)	0.048 (0.345)	-0.043 (0.380)	-0.045 (0.385)
hs-CRP (mg/L)	0.244 (0.042)	0.306 (0.043)	-0.071 (0.513)	-0.089(0.524)
ESR	0.107 (0.459)	0.145 (0.428)	-0.279 (0.032)	-0.344 (0.030)
IL-10 (pg/ml)	-0.070 (0.458)	-0.080 (0.477)	0.072 (0.423)	0.089 (0.410)
IL-6 (pg/ml)	-0.053 (0.407)	-0.064 (0.432)	0.062 (0.317)	0.079 (0.313)
TNFα (pg/ml)	-0.035 (0.642)	-0.044 (0.65)	-0.095 (0.192)	-0.124 (0.185)

1. Kendall's tau-b (*P* value).

2. Spearman’s rho (*P* value).

## Haploview analysis

**Fig 1:**
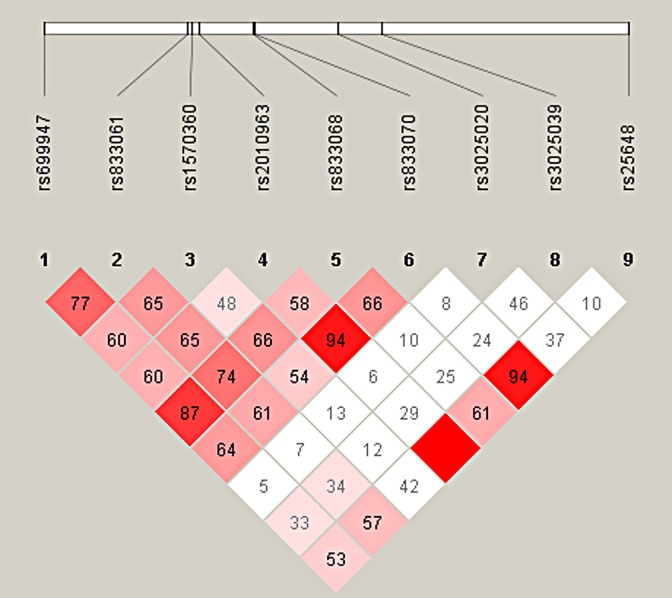
Linkage disequilibrium (LD) map of *VEGFA* SNPs genotyped by Haploview. The positions of the SNPs (Build 37.3) are displayed above the Haploview output. The relative LD between specific pair of SNPs is indicated by the color scheme representing LD relationships, which is based on D’ values (normalized linkage disequilibrium measure or D) multiplied by 100; D’ is calculated as D divided by the theoretical maximum for the observed allele frequencies. Values approaching zero indicate absence of LD, and those approaching 100 indicate complete LD. The square colored red represent varying degrees of LD < 1 and LOD (logarithm of odds) > 2 scores; darker shades indicating stronger LD.

Limited linkage disequilibrium (LD) was noted between the 12 tested *VEGFA* variants, of which 9 were found to be in LD, and thus were used in constructing 9-locus haplotypes (Fig 1). Extensive diversity in the haplotype assignment was seen, with common haplotype (>2% of total) seen in 10 of the identified SNPs (64.8% of total haplotypes). The association was tested after controlling for BMI, free and bioactive testosterone, SHBG, free insulin and HOMA-IR. Increased frequency of haplotype CAACAGCGA (*P* = 0.008) was noted in women with PCOS than control subjects, thus assigning PCOS susceptible nature to this haplotype [OR(95% CI) = 2.91 (1.29–6.88)] (Table 6).

**Table 6:** Haplotype frequencies across the nine *VEGFA* SNPs.

Haplotype ^*2*^	Frequency	Case:Control frequencies	χ^2^	*P*	aOR ^2^ (95% CI)
CACTGCCAA	0.201	0.213, 0.191	0.70	0.40	
CACTGGCGA	0.135	0.157, 0.114	3.49	0.06	
CAACAGCGG	0.103	0.120, 0.087	2.52	0.11	
CTACGGTGG	0.045	0.051, 0.039	0.71	0.40	
CAACGGTGG	0.039	0.042, 0.037	0.11	0.74	
CAACAGCGA	0.032	0.047, 0.015	6.98	0.008	2.91 (1.29–6.88)
CACTGCCGA	0.027	0.019, 0.038	3.08	0.08	
CACTGGCGG	0.026	0.029, 0.023	0.31	0.58	
AAACAGCGG	0.020	0.021, 0.020	0.01	0.95	
CACCGGCAA	0.020	0.026, 0.013	2.20	0.14	

*1*. *VEGFA* block containing rs833052, rs1547651, rs699947, rs833061, rs1570360, rs2010963, rs25648, rs833068, and rs833070.

2. aOR = adjusted odds ratios; variables that were controlled for were BMI, free and bioactive testosterone, SHBG, free insulin and HOMA-IR.

## Discussion

Previous studies that investigated the contribution of VEGF to pathophysiology of PCOS focused on altered secretion/levels of VEGF in women with PCOS, and only few addressed the association between PCOS and *VEGFA* polymorphisms. We evaluated the association of *VEGFA* variants with changes in VEGF serum levels, and the presence of PCOS. Of the 12 SNPs analyzed, we documented the positive association of rs3025020 and negative association of rs2010963 with changes in VEGF secretion and the presence of PCOS. Our study also confirmed the association of CAACAGCGA haplotype with increased risk of PCOS. To our knowledge, this is the first study to show such a link between PCOS and the tested *VEGFA* variants and specific haplotypes.

All subjects were self-reported Arab Bahraini women, who were consecutively enrolled so as to minimize type II errors. PCOS diagnosis was based on the 2003 Rotterdam Criteria, which allows for inclusion of ultrasound examination findings [12, 27]. By comparison to previous related studies, mean BMI was higher in the studied population, similar to the study on South Indian women [20], but different from Korean [28] and Turkish [19] studies, which involved lean PCOS cases and control women. This in turn translated into lower testosterone and insulin compared to our study [19, 28]. Accordingly, BMI, free and bioactive testosterone, SHBG, free insulin and HOMA-IR were the main covariates that we controlled for in this study.

Located on chromosome 6 (6p21.3), the human *VEGFA* gene consists of eight exons and seven introns spanning 14 kb [29], and alternate splicing results in the generation of a family of related proteins [14]. Several polymorphisms dispersed throughout the *VEGFA* gene was reported (NCBI Gene association no: NT 007592), some of which associated with altered VEGF secretion [15, 24]. The latter included the promoter rs833061 (−460T/C), the functional intronic rs3025020 (-583C/T) [24], and 5’-untranslated region rs2010963 (+405G/C), which are relevant to altered VEGF protein expression.

A strong association between increased serum VEGF levels and PCOS was previously reported, and a correlation between serum VEGF levels and increased ovarian stromal blood flow in women with polycystic ovaries was suggested [10, 11, 30, 31]. In our hands, rs3025020 was associated with increased, while rs2010963 was linked with decreased VEGF secretion, in partial agreement with our previous studies on recurrent spontaneous miscarriage [24], and sickle cell vasoocclusive crisis [32]. While not tested here, it is likely that SNPs, in particular those localized in promoter and protein encoding regions may modulate transcriptional events. This does not exclude the involvement of intronic SNPs as cis-acting elements in regulating gene expression [33, 34].

The association between *VEGFA* gene polymorphisms and PCOS was reported for Korean [28], Turkish [19], and South Indian [20] populations, but with mixed outcome. This was attributed to diversity in sample size, ethnic origin of participants, study designs, diagnostic criteria of PCOS, and statistical methods. Neither rs833061 [19, 20] nor rs699947 [19] were significantly associated with PCOS among Turkish [19] and South Indian [20] women. While none of the 10 “common” *VEGFA* variants was associated with PCOS in Koreans, the study of Lee on 134 Korean women with PCOS and control women identified +13553C/T (*P* = 0.033) and +9812 C/T (*P* = 0.044) to be negatively associated with PCOS [28]. It should be noted that the afore-mentioned study included lean women (BMI: cases = 23.22 ± 3.88 kg/m^2^, controls = 20.73 ± 2.36 kg/m^2^), thus questioning the generalizability of the findings.

In this study, rs3025020 (-583T>C) was significantly associated with PCOS in Bahraini women, evident by the enrichment of its minor allele, and homozygous minor allele-carrying genotypes in women with PCOS. Phenotypically, the rs3025020 (-583T>C) polymorphism correlated to increased levels of VEGF in the serum, with the highest levels seen in homozygote carriers, in agreement with our previous study [15]. To a limited extent, rs2010963 (-634G/C) was also found to be negatively associated with PCOS, which was noted in heterozygous genotype carriers. This suggests a possible susceptible and protective nature of these variants, respectively. The lack of association of the other variants, including rs833061 (-460T/C), rs699947 (-2578C/A), and rs1570360 (-1154G/A), were reminiscent of Korean [28], Turkish [19], and South Indian [20] studies. While not tested for its possible association with PCOS, rs3025020 (-583T>C) was previously linked with increased risk of recurrent pregnancy loss in Bahraini [24] and Chinese [25] women. While some of the SNPs included here were previously investigated in relation to other adverse pregnancy complications, including ovarian hyper-stimulation syndrome [35], recurrent miscarriage [24, 25], preeclampsia [22, 23], ovarian cancer [21], and cervical cancer [26] with variable results. As such, our finding on its association with PCOS expands the contribution of rs3025020 to adverse pregnancy complications.

This is the first study to show a significant relationship between the *VEGFA* rs3025020 (-583T>C) and rs2010963 (-634G/C) variants and PCOS. Our study has some strength points, namely that PCOS cases and control women were ethnically matched, thus minimizing the problems of differences in genetic background, which is inherent in genetic association studies, and that several key covariates were controlled for. However, the study has some limitations, principally due to its retrospective case-control design, which can prompts speculation as to the cause-effect relationship. Another limitation is the relatively small sample size, which prevented subgroup analysis of PCOS case, and that only 12 *VEGFA* SNPs were analyzed, thus raising the possibility of the contribution of the other *VEGFA* SNPs (+9812C/T, and +13553C/T) in PCOS pathogenesis. Follow-up studies on populations of related and unrelated ethnic backgrounds, are needed in order to confirm our findings.

## Supporting Information

S1 Data Data available from the Dryad Digital Repository: http://dx.doi.org/10.5061/dryad.nb1hc (XLSX)
